# Evaluating primary care networks in low-income and lower middle-income countries: a scoping review

**DOI:** 10.1136/bmjgh-2023-012505

**Published:** 2023-08-14

**Authors:** Adwoa Agyemang-Benneh, Igor Francetic, Jonathan Hammond, Katherine Checkland

**Affiliations:** Division of Population Health, Health Services Research and Primary Care, The University of Manchester Faculty of Biology Medicine and Health, Manchester, UK

**Keywords:** Health systems evaluation, Public Health

## Abstract

**Introduction:**

Primary care networks (PCNs) are claimed to be an effective model to organise and deliver primary healthcare through collaborative relationships and effective coordination of primary care activities. Though increasingly implemented in different contexts, there is limited evidence on the effectiveness of PCNs in low-income and lower middle-income countries (LLMICs).

**Objective:**

Our scoping review aims to understand how PCNs in LLMICs have been conceptualised, implemented and analysed in the literature and further explores the evidence of the effectiveness of these networks.

**Methods:**

We structured our review using Arksey and O’Malley’s framework for scoping reviews and recommendations by Levac *et al*. We also used the population, concept and context (PCC) guide of the Joanna Briggs Institute (JBI) methodology for scoping reviews to define the search strategy. The identified documents were then mapped, using Cunningham’s evaluation framework for health networks, to understand how PCNs are conceived in LLMIC settings.

**Results:**

We identified 20 documents describing PCNs in five LLMICs. The selected documents showed differing forms and complexities of networks, with a majority resourced by government, non-governmental and donor entities. Most networks were mandated, and established with defined goals, although these were not always understood by stakeholders. Unlike PCNs in developed settings, the scoping review did not identify integration of care as a major goal for the establishment of PCNs in LLMICs. Network evaluation relationships, outputs and outcomes also varied across the five networks in the identified documents, and perceptions of effectiveness differed across stakeholder groups.

**Conclusion:**

PCNs in LLMICs benefit from clearly stated goals and measurable outcomes, which facilitates evaluation. In order to maximise the benefits, careful attention to the aspects of network design and operation is required. Future research work could shed light on some of the missing pieces of evidence on their effectiveness by, for example, considering differential consequences of modes of network establishment and operation, including unintended consequences in the systems within which they reside, and evaluating long-term implications.

**WHAT IS ALREADY KNOWN ON THIS TOPIC**

- Primary care networks are intended to facilitate the effective orchestration of care by coordinating the activities of providers at different levels in a health system.
- Trusting collaborative relationships are expected to increase resource access and improve governance.

**WHAT THIS STUDY ADDS**

- Primary care networks in low-income and lower-middle income countries are often mandated and goal-oriented, although integration of care does not appear to be a major area of focus. They are also varied in complexity, and though there is some description of types of networks implemented, evidence on their evaluation relationships and output/outcomes is limited.

**HOW THIS STUDY MIGHT AFFECT RESEARCH, PRACTICE OR POLICY**

- Policymakers may benefit from designing and engineering primary care networks using frameworks that allow for evaluation at all stages of implementation. Policymakers may also do more to involve community members as stakeholders in network development and operation. Gaps for future research are identified.

## Introduction

Primary healthcare (PHC) is lauded as a stable way to maintain a health system over time, and a cost-effective approach to organising and delivering healthcare.^1^ PHC is thus a foundational element for the provision of universal health coverage,^1^ and since the Alma-Ata declaration in 1978, many health systems in resource-constrained settings have focused on optimising the delivery of primary healthcare.^2^

An increasingly popular mode of primary care delivery is the primary care network (PCN). The network model is viewed as a way for policymakers to purposefully bring together varied actors to collaborate around a common goal, defining the mechanisms by which they function, are structured and governed.^3^ While public networks are often mandated to solve complex problems,^4^ networks in healthcare are usually a way to organise service delivery and/or governance structures.^3 5^ In service delivery, networks are aimed to prioritise patient-centred care and efficient management, through collaborative activities of varied actors, including community members.^6^ It is also argued that networked models of service provision allow the effective orchestration of care by coordinating the activities of providers at different levels in the system and building trust, thereby increasing access to resources, and improving governance.^3^

For our study, we draw on the above definitions of healthcare networks to outline a working definition of primary care network as a model of community-based health service delivery and/or governance arrangement proposed and implemented so multiple primary care actors may coordinate around a common goal(s). A PCN is typically a mandated network form where policymakers decide on the need and purpose of the network and the timing of its creation, and set the parameters for network membership, structure and access to new or reallocated resources.

There is an extensive literature on PHC programmes in low-income and lower middle-income countries (LLMICs) but, despite their increasing popularity,^7 8^ the conceptual understanding of networked models of governance and delivery of primary care is limited. To address this gap, this scoping review identifies how primary care networks in LLMICs have been conceptualised, defined, and analysed in the literature.

## Methods

### Rationale

We build on the Arksey and O’Malley^9^ framework for scoping reviews and recommendations by Levac *et al*^10^ to structure our review and guide the discussion of our findings. We also used the population, concept and context (PCC) guide of the Joanna Briggs Institute (JBI) methodology for scoping reviews,^11^ to define the search strategy and the inclusion and exclusion criteria. We opted for a scoping review instead of other systematic approaches in order to effectively map the literature to understand how PCNs are conceived in LLMIC settings. A scoping review provides a broad, comprehensive overview of a literature, and helps to summarise evidence available, as well as identify gaps.^12^ Scoping reviews also help to incorporate varying sources of literature, including policy documents.^11^

### Eligibility criteria

Using our definition for PCNs, and the PCC guide, we defined the following inclusion criteria for our scoping review:

- Population: All citizens/residents of LLMICs. Studies conducted on populations in high and upper middle-income countries were excluded.
- Concept: PCN/healthcare network/networks of care. Networks that existed solely between individual members of the health workforce, or at the secondary and tertiary levels of the healthcare system alone were excluded. Documents on research, surveillance or telemedicine networks were excluded. Documents that discussed models or policies of PCNs that had not been implemented were excluded. Documents on specific diseases or programmes at the primary care level were also excluded.
- Context: LLMICs according to the World Bank classification of countries by income level as of 2022.^13^ We explicitly exclude upper middle-income and high-income countries to restrict our review on health systems with more comparable resources. While governance structures may be similar, the resources available for health systems of countries in the upper-middle income group are substantially different and comparing them to low-income countries could provide a confounded picture. This is in line with other recent review work^14^

All forms of literature, including peer-reviewed articles and grey literature, were included, but documents were excluded if the completed work could not be successfully retrieved. Thus, some literature, such as conference abstracts, were excluded from the review. No time or language restrictions were used in the search strategy.

### Search strategy

The focus of the scoping review was to identify PCNs established in LLMICs. Keywords included ‘primary healthcare’ OR ‘primary care’ and ‘network OR networks’ in conjunction with names of all countries categorised as low-income and lower middle-income according to the World Bank income groups classification for 2022.^13^ We initially searched two databases, MEDLINE (PubMed) and Embase (Ovid), using the above keywords in November 2022. The search was repeated in May 2023 in Health Management Information Consortium (HMIC) and Global Health (Ovid), using the above keywords, but yielded no new results. The full database search is presented in online supplemental file 1. After search results were de-duplicated using reference manager Zotero, title and abstract screening was manually conducted by AA-B. Full articles were then retrieved and reviewed by AA-B and IF for inclusion using the inclusion/exclusion criteria detailed above.

10.1136/bmjgh-2023-012505.supp1 Supplementary data

The reference lists of identified documents were further searched for relevant titles using ‘forward snowballing’ by AA-B and IF. With consideration for the eligibility criteria, the relevant titles were searched on Google Scholar and other national and international healthcare websites. This was done by entering the full title of the relevant text into the search engine of the website and retrieving the full text, where available. The full list of websites searched is presented in online supplemental file 2. These websites were also searched using the keywords to identify any grey literature relating to PCNs in LLMICs. Returned results were manually subjected to the eligibility criteria and added to the final selected documents for data extraction.

10.1136/bmjgh-2023-012505.supp2 Supplementary data

The data extraction was conducted by AA-B and IF and validated by JH and KC. Data were extracted according to the parameters identified from the evaluation framework.

### Framework for healthcare network evaluation

**Figure 1:**
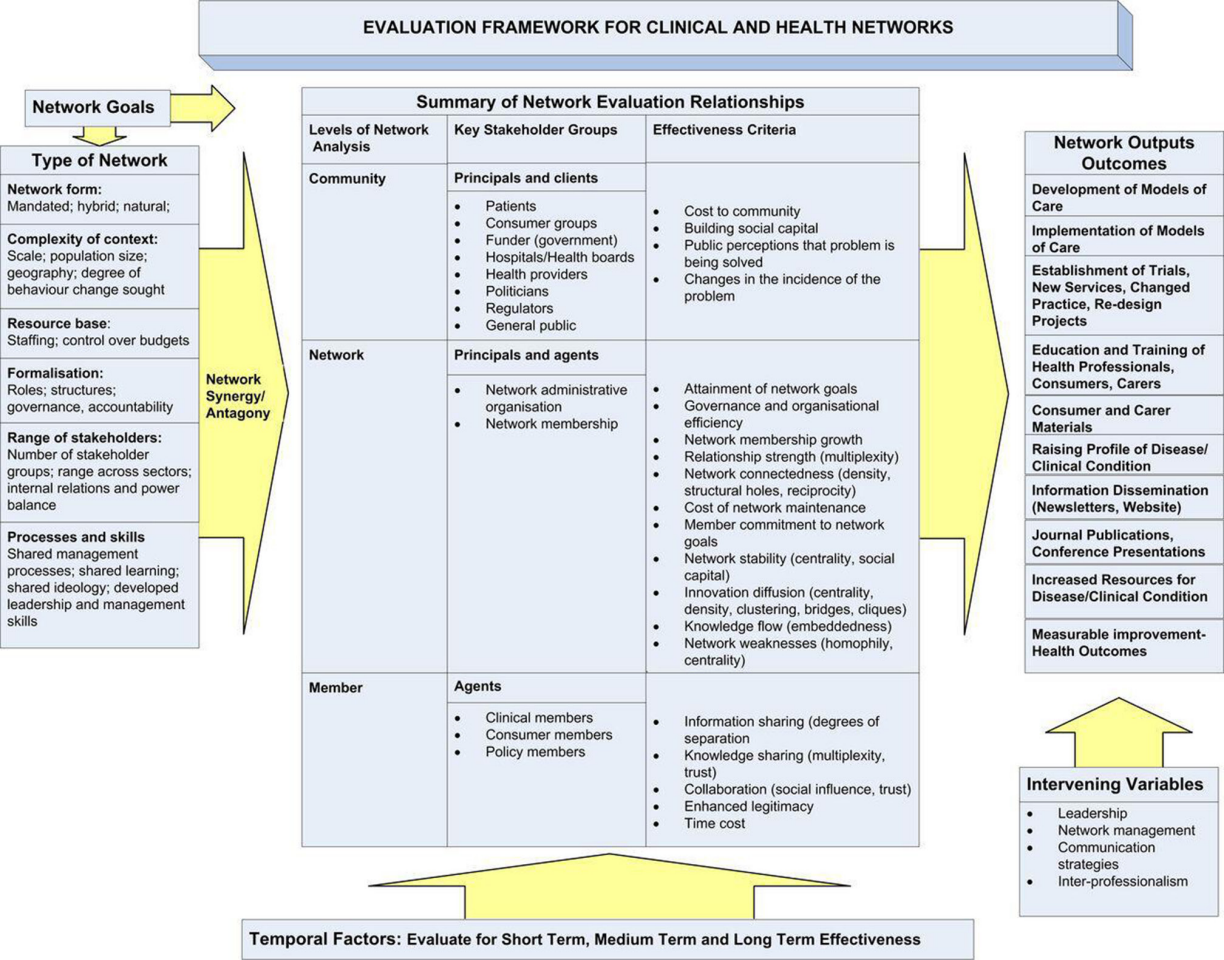
Evaluation framework for clinical and health networks.^15^

Cunningham *et al*^15^ developed an evaluation framework for clinical and healthcare networks based on a systematic literature review and with input from a diverse expert advisory group (figure 1). The framework aligns with the recommendation by Provan and Milward^16^ that community-based health networks with multiple stakeholders should be evaluated comprehensively, considering the different levels involved, to determine whether a network is achieving its purpose or not.

We used this framework to organise and explore the literature identified from the search. For each LLMIC context, we identified the goals for network implementation, including network characteristics such as form, context, resource, stakeholders and processes. Behaviour change sought and network governance were also explored under network characteristics.

We then considered intra-network collaboration as well as collaboration with their external partners across three main levels: community, network and member. Network outputs and outcomes were also evaluated based on these three levels. The evaluation framework suggests a number of intervening factors, including leadership, management, communication and interprofessionalism are also important,^15^ and these were further explored.

Finally, Cunningham *et al*^15^ emphasise temporality as an important factor affecting network effectiveness and recommend that a network should be evaluated with sensitivity to the stage of its implementation. Thus, evidence on short-term, medium-term and/or long-term effectiveness were explored. Network goals, sustainability and viability and innovative capacity were also assessed.

### Patient and public involvement

No patients/public were involved in this study.

## Results

### Search results

**Figure 2:**
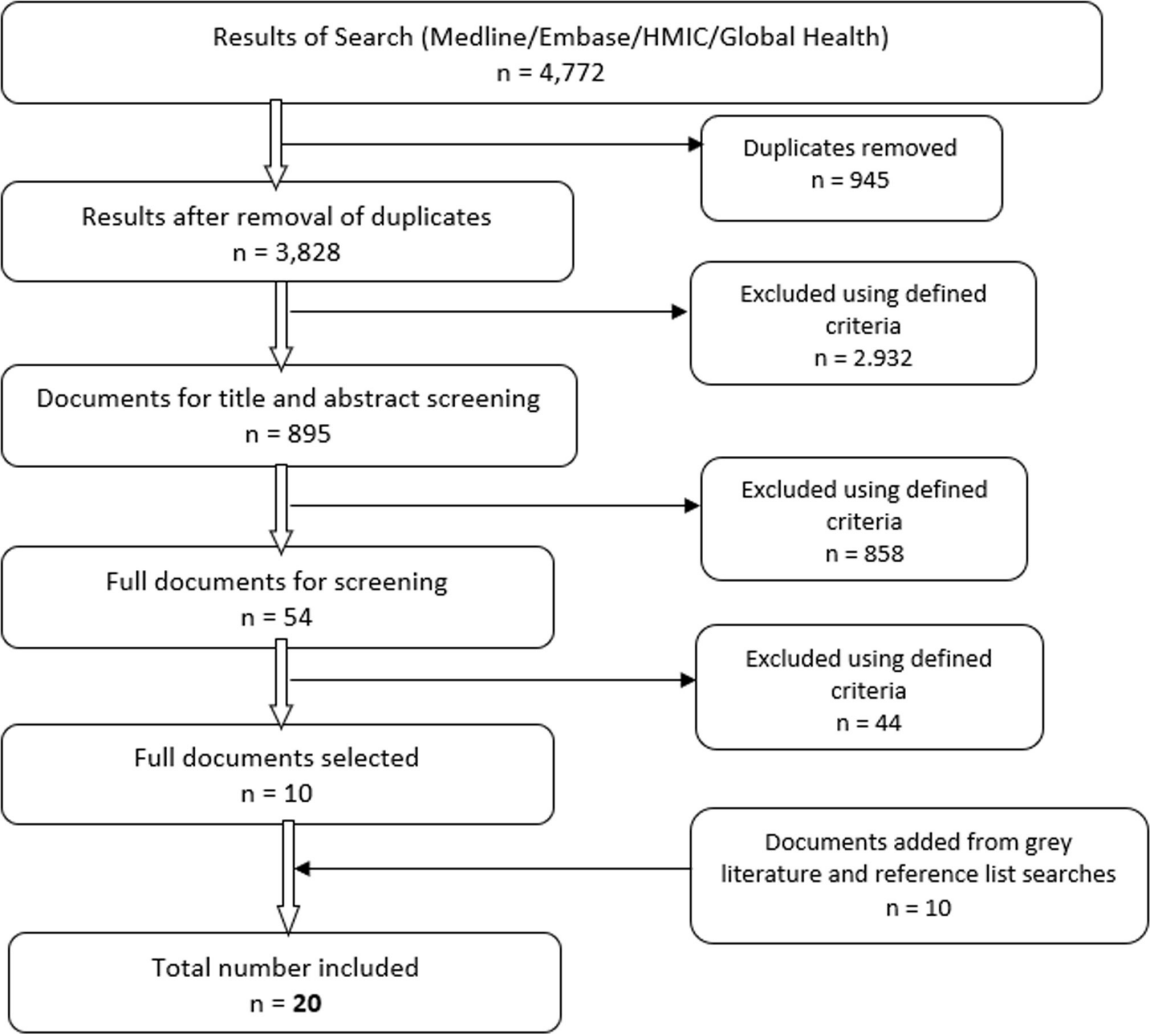
Preferred Reporting Items for Systematic review and Meta-Analysis flow chart of search results. HMIC, Health Management Information Consortium.

The initial database search returned a total of 4772 results. After removal of duplicates (n=3828), title screening (n=993) and abstract screening (n=895), 54 documents were retrieved for full-text screening. Ten documents were included in the review after screening, and an additional 10 were discovered through hand searches of reference lists of the screened documents, generating a total of 20 documents. The only non-English document (in Spanish) was initially translated using Google Translate. The translation was then reviewed and corrected by a colleague fluent in Spanish, prior to data retrieval. Figure 2 represents the screening process according to the adapted Preferred Reporting Items for Systematic review and Meta-Analysis framework for scoping reviews.^9^

We identified documents on primary care networks in five LLMICs: Iran (11), Lesotho (4), Democratic Republic of Congo (DRC; 2), Honduras (2) and Bolivia (1). Online supplemental file 3 includes a detailed description of the included documents.

10.1136/bmjgh-2023-012505.supp3 Supplementary data

### Analysis of search outcomes

The identified documents were analysed under three main headings, consistent with Cunningham *et al*’s^15^ framework: network goals and characteristics; network evaluation relationships; network outputs/outcomes. A summary of this is contained in tables 1–3 and the implications explored in the subsections below.

**Table 1:** Network goals and characteristics

Network goals						
		Iran	Lesotho	DRC	Honduras	Bolivia
Network goals		To improve access to public healthcare in disadvantaged areas and reduce gaps between rural and urban areas in terms of health service delivery. To improve quality of healthcare services.	‘To achieve better outcomes by contracting out the building and operation of an integrated health network, increase accountability for service delivery and quality of care through a performance-based contract, while maintaining the government’s important role as steward of the health sector.’^25^	Improving geographical access to healthcare and achieving socioeconomic sustainability.	In general: to develop health systems based in primary care and the delivery of equitable and efficient high-quality health services that better meet people’s expectations. Individual networks may have additional goals.	Ultimate objective of the pilot is to reduce maternal and child mortality in the country. Supply driven goal: improve quality and efficiency of healthcare services.
**Network characteristics (type)**						
Network form		Mandated	Mandated	Mandated	Hybrid (depending on structure of network)	Mandated
Complexity of context	Scale	National	Implemented in one district as an 18-year funded project.	National. Country is subdivided into 515 health zones.	National, but article describes five networks.	Implemented as a pilot in one municipality.
	Population/ geographical coverage	Rural (7500), urban (12 500). Health houses cover population of about 1500.	About 25% of country’s population; major referral hospital with four community-based clinics.	Health centre: 5000–10 000, health zone; 110 000.	Article covered networks with more than one municipality and in areas of high poverty. Population size not stated.	140 000 (a quarter of population of El Alto municipality).
	Behaviour change sought	Bridge gap between rural and urban healthcare.	Build community trust in local (primary) healthcare system.		Move from highly fragmented health system to integrated networks.	Strengthen first level of care and develop efficient referral and counter-referral system within network.
Resource base	Staffing	Government led. But community health workers recruited from local community.	Done by private partner.	NGO recruited healthcare workers.	Health teams as the first level of care. Recruited based on structure of network.	NGO recruited healthcare workers.
	Control of budgets/funding	Government-funded networks. Health is also financed by various insurance schemes such as Social Security Organization, Medical Service Insurance Organization and Emdade-Emam Committee Health Insurance.	Public–private partnership, where a private entity runs the entire network and the central government reimburses the network through a contract with predetermined goals/standards and penalty mechanisms. Funding was supported by World Bank Group.	Health zones operating with autonomy but with networked governance structures to the central government. Official agreements between state and non-state partners also exist.	Decentralised networks have management agreements with negotiated allocations. Some have explicit and strategic incentive mechanisms to reach network goals.	Management contract between government and NGO.
Formalisation	Governance	Provincial medical university responsible for governance in each district.	Led by private entity, with supervision of network by local government.		Three types of network governance. Decentralised: led by the local governments and NGOs. Non-decentralised: led by central government. Mixed: managed by a combination of authorities.	Management contract committee responsible for contracting and purchasing. Minimum of two community representatives.
	Structure	Each rural/urban health centre supports up to five health houses/health posts or health bases, forming a PHC network, administratively supported by district health centre, clinically supported by district hospitals.	Four primary care clinics networked to one national referral hospital.	Health zones with health centres and central health zone office.	Health teams with network coordinators and supervisors.	One district hospital as hub with eight health centres as spokes. Supervised by management contract committee (as the buyer) and NGO (as the provider).
	Accountability	Each district health network is accountable to the provincial medical university within that district.	Independent monitor to assess project performance against contractually agreed clinical and non-clinical indicators.		Results-based management in networks. Annual operating plan with measurable objectives spelt out in decentralised networks.	Results-based management.
Range of stakeholders		Communities, local government, healthcare providers.	Government, donor (World Bank Group), private consortium.		Decentralised networks operate independent of central government entities. Official documents do not highlight mechanisms to enhance social participation, but some evidence of this exists at the practical level.	
Processes and skills:	Shared management process	Involvement of communities and local government in rural health system decisions.	Management responsibility lies with the private consortium but the government is in charge of supervision and accountability processes.			Management process: Involvement of two community members in contracting committee

DRC, Democratic Republic of Congo; NGO, non-governmental organisation; PHC, primary healthcare.

**Table 2:** Network evaluation relationships

Network evaluation relationships						
Key stakeholder groups						
Levels		Iran	Lesotho	DRC	Honduras	Bolivia
Community	Principals and clients	Community health workers (*behvarzan*), health technicians, midwives, physicians, hospital board of trustees.	Government, private healthcare consortium, external funder, independent monitor.	Government, private non-profit organisations, private for-profit organisations, external funders and the population.	Decentralised networks operate independent of central government entities. Official documents do not highlight mechanisms to enhance social participation, but some evidence of this exists at the practical level.	Management contract committee, healthcare workers.
Network	Administrative Organisation	Representative of Ministry of Health and Medical Education heads District Health Network.	Network operations transferred from government to private entity in project.		Could be led by local governments and NGOs, central government or a combination of authorities.	Network operations transferred from government to NGO in pilot.
	Membership	PHC network+district health centre+*behvarz* training centre. Coordinated by District Health Network.			Health teams, network coordinators and supervisors.	
Member	Clinical members	Community health workers (*behvarzan*), health technicians, midwives, physicians.	Healthcare workers.		Healthcare workers.	Healthcare workers.
	Consumer members	Health volunteers.				Community members on purchasing committee.
	Policy Members	Representative of Ministry of Health and Medical Education heads District Health Network.				Mayor, Municipal council.
**Effectiveness criteria**						
Community	Public perception that problem is being solved		Introduction of peripheral local clinics changed patient behaviour towards local health services and built trust in these services.			Introduced mechanisms to reduce waiting time in facilities. Set up customer defence office.
Network	Attainment of network goals	Reports of reduced infant and child mortality, improved maternal health and major decline in infectious childhood diseases.			Decentralised networks had higher scores in evaluation, indicating their goals were somewhat met.	
Network membership growth			Government plan to increase number of healthcare facilities and to set up at least one more PPP network arrangement.			Plan to scale up network model in other parts of the country.

DRC, Democratic Republic of Congo; NGO, non-governmental organisation; PHC, primary healthcare; PPP, public–private partnership.

**Table 3:** Network outputs/outcomes

Network outcomes/outputs	IRAN	Lesotho	Honduras	Bolivia
Development of models of care	Introduction of family practice programmes to enhance primary healthcare networks.	Introduction of PCN highlighted the need for nationwide improvement of the public health system. Similar models of care to be established based on success of this.	Though patient-care is highly prioritised in theory for implementing networks, this high priority was not evidenced in practical assessment of established networks.	More public-led networks planned as public-run facilities are more common in the country.
Education and training of healthcare professionals, consumers and carers.	Incorporated medical training into the health ministry. District-based training centres for community health workers (free). Community-oriented medical education council established.			Training of healthcare providers for change in mentality.
Establishment of trials, new services, changed practice, redesign projects	Health information system which is fed with detailed community-specific information by behvarzen.	Better quality of care, access to advanced healthcare technology.		Development of care standards and protocols for all services offered within the network.
Increased resources for clinical condition/disease	Integration of management of NCDs at the community level			
Measurable improvement (or not)	Rapid improvement of health indices over the years, reduction in maternal and infant mortality, improved equity in rural areas to basic healthcare services Some studies indicate Initial improvements not maintained in the longer-term.	Improved maternal and child health indices in report, health network efficiently treating more people than previous system.		

NCDs, non-communicable diseases; PCN, primary care network.

#### Networks goals and characteristics

##### Network goals

Quality improvement, reducing health inequity and responsiveness were some of the goals identified in network implementation in Honduras and Iran.^17 18^ Networks in DRC and Iran also aimed to improve accessibility of healthcare services^19 20^ and in the former to also achieve socioeconomic sustainability.^20^ The PCN in Bolivia aimed to achieve better health outcomes including reduced maternal mortality, while the Lesotho public–private partnership (PPP) network aimed to improve quality and efficiency of healthcare services.^21 22^

##### Network characteristics (type)

Most networks identified were mandated and established either by a government initiative or through a public–private partnership.^21–24^ In Bolivia and Lesotho, networks were implemented at local level, with a central facility linked to community-based centres.^21 25^ In Iran and DRC, there was national implementation of networks at the district or provincial level.^26 27^ Networks in Honduras were also implemented at a provincial level, as part of the national integration of health service delivery networks.^17^

In terms of size, most networks were established in districts, with emphasis on community-based care, serving population sizes of between 7000 and 140 000 individuals.^20 21 24 26^ In Iran, complexity of the network structure resulted in network members having large geographical coverage in urban areas.^28^

The degree of change sought varied between networks. While PCNs in Iran were described as bridging the gap of healthcare access between the rural and urban areas and bringing healthcare to ‘where people live’,^18 28^ the Lesotho network sought to improve the community’s trust in local healthcare services.^22^ The networks in Honduras sought to move from a fragmented healthcare system to one with integrated networks^17^ and the Bolivia network aimed to change the referral structure and thus strengthen primary care.^21^

Resource availability was linked to the type of partnership (sole or interagency) that led to the network implementation. Regarding staffing, Iran’s networks saw government-led recruitment of staff, including locally recruited community health workers^19 26^ although there was a shortage of staff directly supporting network goals in some cases.^28^ Both DRC and Bolivia had network staff recruited by non-governmental organisations (NGOs)^20 21^ and Lesotho had network staff recruited by the private partner.^25^ For networks in Honduras, staff were recruited by local governments and NGOs (in decentralised networks), central government (in non-decentralised networks) or a combination of authorities (in mixed networks).^17^

Budgetary control was in some cases led by government appointed officials, such as in Iran^29 30^ or enacted through contractual agreements between government and non-governmental entities in the other four country examples.^17 20–22^ For Iran, this budgetary control was sometimes disproportionate between managers at the network headquarters and managers at health facilities.^28^

Structures for network governance were clearly defined. The networks in Honduras, Iran and DRC had hierarchical governance structures that mirrored the structure of the respective health systems.^24 27 31^ PCNs in Lesotho and Bolivia also had hierarchical systems but included an additional hub-and-spoke structure within the network.^21 22^ In addition, the Bolivia network also had a management contract committee which, together with healthcare providers, was responsible for network governance.^21^

Focusing on accountability, the Lesotho network included an independent actor responsible for monitoring network indicators in line with the agreed contract.^22^ In Honduras, decentralised network governance structures evidenced accountability through annual operating plans.^17^ In Iran, accountability structures followed a hierarchical structure, with health houses/posts reporting to health centres who in turn reported to the district hospital and health centre.^26^ However, one paper argued that there were many challenges, including unclear scope of accountability, responsibility and authority, heavy administrative burdens and weak monitoring systems.^28^

Healthcare providers and local or national government entities were key stakeholders in all the included PCNs. All but the Iran PCNs evidenced some form of non-governmental stakeholder group, including faith-based organisations in DRC,^20^ foreign donors in Lesotho^22^ and other not-for-profit agencies in Bolivia and Honduras.^17 21^ In terms of community stakeholders, the Bolivia network was explicit about the inclusion of community members in the management team^21^; while Iranian networks emphasised the inclusion of locally recruited community health workers in the workforce.^26^

Evidence regarding power dynamics affecting PCNs was difficult to discern from available sources. However, in Iran, there was a focus on financial, administrative and decision-making power in district network managers and higher authorities, with a suggestion of inadequate power available to managers of health facilities.^28^ In DRC, it was stated that external non-state partners may introduce vertical programmes that do not harmonise with the wider healthcare strategy.^20^

Shared management processes were described in three of the networks. Evidence from Iran indicated some initial involvement of communities and local government in rural health system decisions, such as selection of local members for training as community health workers^26^ but the multiplicity of new programmes, activities and processes increased the complexity of one network’s structure.^28^ Lesotho evidenced shared processes between the government (funding, supervision and accountability) and the private consortium (health service delivery and maintenance).^22^ In Bolivia, shared management was between the healthcare provider and the purchase committee, through a management contract.^21^ In Honduras, the style of service management, that is, whether the network is decentralised, non-decentralised or mixed, is determined if there were shared management processes or not.^17 24^

Evidence regarding shared learning, shared ideology and developed leadership and management skills was not identified.

#### Network evaluation relationships

Following the evaluation framework described in section **Framework for healthcare network evaluation**, we delineated the networks across three main levels of evaluation relationships: community, network and member. This allowed us to identify the different key stakeholder groups and consider what may or may not constitute effective network operation at these levels. Table 2 shows the network evaluation relationships identified in the screened documents.

##### Community level

Health networks in Iran had health workers, health volunteers, government representatives, provincial medical universities, medical insurance entities and rural dwellers as principals and clients.^18 30^ Public perception of health houses in networks was seen as positive and these were often the first points of contact for clients because of familiarity of locally recruited health workers.^26 31^ Further evidence suggested that family physicians introduced in health centres improved quality of care with prompt referral, and also provided long-term relationships between the population and the healthcare workforce, especially in rural areas.^19^ However, one report suggested that social capital had reduced over the years and the public had a cautious regard for free healthcare programmes of the primary care networks.^32^ Thus, in many urban areas, people sought and paid for healthcare directly at higher levels bypassing local primary care units.^30 33^

In Lesotho, there was no explicit mention of other principals at the community level beyond the health provider, the government and the external funder, and there were mixed public perceptions of the network’s effectiveness. On one hand, McIntosh *et al*^22^ argued that the PPP network was providing vital, quality healthcare to a quarter of the country’s population. However, Webster^34^ argued that there was unequal distribution of resources by central officials, with one-third of the country’s health budget directed towards one health network. The different reports also came to similarly different conclusions about the extent to which health outcomes had improved.^22 34^

For DRC, the different governance structures resulted in health zones with different stakeholder groups. In areas (mostly rural) with few public healthcare facilities, faith-based organisations were key stakeholders.^20^ These organisations built social capital in the community through their healthcare interventions, creating a sense of communal ownership.^20 27^ One study highlighted concern about the apparent absence of the state in community healthcare.^20^

There was insufficient information to evaluate networks in Honduras and Bolivia at the community or network levels. However, Lavadenz *et al*^21^ mention the introduction of mechanisms such as a customer defence office and a facility-based system to reduce waiting time in Bolivia, which may contribute to improved public perceptions of healthcare delivery.

##### Network level

At the network level in Iran, the district health network team, with Ministry of Health and Medical Education representatives, was responsible for network administration. Networks consisted of primary healthcare delivery facilities networked with provincial medical schools and training units for community health workers.^18^ Iran’s PCN achieved some intended goals in the early stages of the policy, but, more recently, these have stalled due to various factors including epidemiological shifts in disease patterns and rural–urban migration.^32^ The burden of inadequate primary healthcare was felt more in urban areas, where networks were underdeveloped and comparatively less efficient, and where clients had direct access to secondary and tertiary facilities.^32 35^ Though some argue that the governance structure in Iran resulted in increased organisational efficiency,^26 31^ others suggest that the lack of accountability, supervision and quality control mechanisms for human resources and other components of health service delivery affected the efficiency of the PCNs.^18 30^ Furthermore, in Iran, tasks and responsibilities assigned to health networks appeared to be influenced by political, economic, social, and environmental changes.^28^

In Lesotho, the network’s administration was shared between the government and the private consortium, with a closed membership. The network attained its goal of improving health outcomes through a PPP,^36^ but effectiveness of accountability structures was unclear.^34^ In addition, improved healthcare in the area served by the network may have increased service demand, increasing operational costs. This reportedly strained relationships between public and private actors when the cost of the network exceeded contractually agreed limits.^25 34^

Little information was available on effectiveness at the network level for DRC, although there was consensus regarding insufficiency of healthcare availability.^27^ It was generally perceived that faith-based facilities frequently provided better healthcare than government-run health zones.^20 27^ Again, the relationships between network agents were often weak, and mistrust between some state and non-state agencies was reported.^20^

##### Member level

In Iran, effectiveness of networks at the member level was mixed. Volunteers and community workers collected key demographic and health data from the community and submitted these to policymakers through network governance structures, supporting information and knowledge sharing.^29 30^ Training and supervision were also provided to healthcare workers through the district health centre, district hospital and training institutions.^18 26^ However, this training was not always tailored to the needs of the population, resulting in an inefficient and demotivated workforce.^33 35^ The organisational structure also did not always align with the expectations of staff, leading to reduced motivation.^28^ Collaboration between members of the network was also sometimes inefficient, with challenges around timely information sharing.^35^ Though collection of data from communities was a core function of community health workers, inefficiencies in the data collection process caused this to be viewed as a waste of time.^35^

The identified documents did not include information relating to the effectiveness criteria at the member level for networks in Lesotho, Honduras, Bolivia, or DRC.

#### Network outputs/outcomes

The identified documents provided limited details on the outcomes and outputs of PCNs. Where available, this evidence often lacked any objective assessment of claims made about network effectiveness or otherwise. Crucially, the documents available for the PCN in DRC did not include any useful details on this aspect of PCNs. Table 3 below shows a summary of the outputs and outcomes identified in the documents reviewed.

#### Synthesis of results

The main purpose of this study was to explore what evidence there is about how primary care networks are described and implemented in LLMIC rather than attempt to draw generalisable lessons, as would be typically the case for systematic review. We chose this approach because this is a relatively new topic, and because it is not yet clear whether there is sufficient high quality evidence available to allow the posing of more specific questions which would usually accompany a systematic review.^37^ As such, our cross-study synthesis focuses on what is needed if future evaluations and studies are to be useful in addressing wider questions about the value and impact of the introduction of primary care networks as a means of delivering primary healthcare services.

All identified networks had evidence of clear goals for why they were established. Characteristics of the type of network were also identified for the majority of networks. However, evidence on roles of the network (formalisation), range of stakeholders involved and processes and skills of the network were largely unavailable in the identified literature.

For evaluation relationships, the evidence was sparse across all levels, especially for the network and member levels. Very few identified documents touched on the effectiveness criteria of these relationships. Features such as network connectedness, relationship strength, innovation diffusion, degrees of separation, collaboration and innovation flow, among others, were also largely missing. These are key areas to explore because they highlight the importance of the network concept, and the relevant factors to be considered when a network approach is employed. Importantly, the documents reviewed also did not show explicit involvement of community members in networks, aside being recipients of the service, which is critical for the effectiveness of any primary care model.

Finally for network outputs and outcomes, most networks identified developed new practices after introduction of the network model, but information was limited. Effectiveness of networks was implied for almost all the networks identified, but the evidence presented did not give an objective or conclusive indication of this.

Evidence on temporal factors (except for networks in Iran) and intervening variables, such as leadership and interprofessionalism, were also unavailable in the documents explored.

## Discussion

This scoping review shows that the PCNs in LLMIC were mostly focused on improving quality, equity and responsiveness in health service delivery to achieve better health outcomes. This illustrates that, in LLMICs, there has been an effort to define a goal for the networks, with some stated reasons for their establishment. This is consistent with the evidence that networks in healthcare are frequently deliberate governance structures introduced to support different actors to work towards a common goal.^3^ The stated goals, however, were not always clear, agreed on or understood by all stakeholders and network members. Unlike in developed settings, the scoping review did not identify integration of care as a major goal for establishment of PCNs in LLMICs. This finding may reflect an implicit aim, among the networks identified in our review, to strengthen primary healthcare services, as a means of achieving universal health coverage. Therefore, the main goals of the network approaches covered here focused on extending the provision and quality of care rather than the integration of existing care services.

Additionally, the identified documents do not explain why a network approach was adopted in any of the identified countries. There is no indication that other potential PHC models were considered or any evidence detailing a selection process featuring alternatives.

### Network type

In four out of the five networks discussed in this paper, there was a government-led directive to implement the network concept, with evidence of goal setting and some prescription of membership and governance structure. Thus, most of these networks can be characterised as mandated, which is typical of public sector networks.^4^ Government involvement was significant in the administrative and financial oversight of the networks, but there was a notable dependency on non-governmental actors in many countries. This is common in LLMICs, with a substantial presence of external partners such as foreign agencies, donor or technical partners and religious bodies.^38^ This may make the network concept particularly appealing, as networked organisational forms are perceived to be a way of bringing together diverse stakeholder groups to cooperate, mobilise resources and coordinate efforts towards achieving common goals.^39^ However, they may also make them more difficult to implement, as external donors may have objectives which do not fully align with those of the relevant government.

Where the information was available, the review showed that network structures were typically developed to mimic existing PHC structures. This is consistent with evidence from Provan and Kenis^40^ who highlight that network governance forms are typically based on existing structures or imitation of other networks. Basing networks on existing structures may be a way to ease network members into a new mode of governance through a familiar system, but it invites consideration as to whether there is a need for a new network approach at all.

The network structures included in this review were either partially hierarchical or hub-and-spoke models. In these structures, accountability was often from network managers (ie, health workers and implementing agents) who answer to the government and non-governmental entities or independent actors often at a higher organisational level. These structures were similar to lead organisation-governed networks where one network member takes up the leadership role based on the member’s centrality.^40^ Healthcare systems traditionally rely on hierarchical governance, with clear reporting and supervisory lines,^41^ making other features of networks, such as shared participant governance, infrequent in public healthcare systems. However, these centralised governance systems may introduce power imbalances that might militate against a sense of ownership for all members. Thompson and Thompson^42^ characterise networks as having equal membership and Popp and Casebeer^3^ state that centralised leadership may undermine members’ sense of shared risk and legitimacy. Furthermore, network governance ideally provides some decision-making autonomy to network managers,^43^ but this may not hold true if there is a ‘superior’ unit that strictly regulates managers’ activities. In LLMICs where PHC is delivered primarily by community health workers, the power imbalance between these and the higher health workforce categories may be detrimental to the collaborative intentions of networks. In summary, this review did not find clear pathways by which PCNs can accommodate the multilayered nature of primary healthcare governance. We found a particular tension between the reliance of agents at the service delivery level in public facilities on higher administrative levels of the government for resources and regulations, and their need to respond to local communities.^44^

### Evaluation relationships

#### Community level

The identified documents suggest a lack of representation of community members as key stakeholders within networks or specific mechanisms by which network members are accountable to the communities they serve. Information on processes and skills of networks was also limited. The evidence also highlights a need for more emphasis on community involvement in network implementation and on the direct impact of networks on social capital, public perceptions and cost to the community. A community’s perception of a network’s effectiveness directly affects how acceptable network services are to the community members.^15^ Thus, network implementers should consciously include and evaluate network effectiveness at a community level to better appreciate the overall impact of these networks. Iran’s primary care networks had the broadest group of stakeholders at community level, but public perception of the networks and perceptions of cost to the community evolved over time. Cunningham *et al*^15^ highlight the temporality of network effectiveness, indicating that policymakers must continually evaluate and adapt network governance and processes in line with developments over time.

#### Network and member levels

In evaluating effectiveness at the network level, most countries employed a lead-organisation governance structure, mostly led by a public entity. Some effectiveness criteria appeared to be met, with networks achieving their intended purpose, but accountability, relationship strength, network connectedness and network stability were not sufficiently explored in the identified documents. Member level analysis highlighted training and supportive supervision for the health workforce within the network, and knowledge and information sharing between different levels of the network. Again, community members were largely absent. Opinions on the effectiveness of Lesotho’s network were varied, depending on the viewpoint of the stakeholder concerned. In DRC, networks in areas manned by non-governmental agencies were seen as more effective than in areas without NGOs, and the public perception was that the government was ‘absent’ in healthcare provision.^20^ The situations in Lesotho and DRC may indicate the lack of multisector stakeholder engagement, especially in relation to community and civil societies.

Although trust is critical to the effectiveness and sustainability of networks, no evidence was found of it being cultivated or assessed in published reports. Provan and Kenis^40^ note the need to understand the distribution and reciprocity of trust within a network to guide decisions on the appropriate governance structure. The type of governance structure adopted should reflect these factors rather than mimic existing structures.^3^ Popp and Casebeer^3^ also mention that network implementers must address power differentials and perceived trustworthiness, among others, to build trust into a network and hence improve its effectiveness. These are resource-intensive activities which should be incorporated into resourcing of network activities.

Assessing the network structure and evaluation relationships in the identified documents, we argue that the use of the term ‘network’ often conveyed the impression of introducing a new organisational model, even though the existing system remained largely unchanged. This, coupled with the fact that key relationships and elements of healthcare networks such as knowledge flow, relationship strength and collaboration could not be found in the identified documents, may be an indication that even though the term network is used, it does not necessarily indicate that a network governance form is being practiced. Such healthcare collaboratives may be seen as ineffective networks, even though they have not been adequately conceptualised and implemented as such. They may find it difficult to achieve their goals because they are not operating in a manner consistent with a network approach.

### Outputs/outcomes

The network analysis framework employed combines network outputs (what the network does) with network outcomes (immediate gains),^15^ making it difficult to analyse these two distinct features for networks in LLMICs. The evidence available, however, identified some measurable network outcomes, notably in Iran^26^ and Lesotho,^22^ suggesting the network approach potentially improved patient care and health workforce coordination. This cannot be a conclusive observation, however, as the success of an intervention may be an effect of the setting within which the intervention is introduced, and not necessarily a direct consequence of the intervention itself. Overall, the identified documents do not provide sufficient breadth of information to critically discuss network outputs and effectiveness in LLMICs. In the case of Lesotho specifically, the network was simultaneously considered effective and ineffective depending on the perspective presented. Perceived effectiveness of Iran’s networks was also questioned depending on the time the study was done. Thus, to fully evaluate network effectiveness, it is important to explore varying perceptions of their implementation and the resultant consequences (intended and unintended) as well as consider the temporal factors at play.

### Challenges in applying the evaluation framework

The evaluation framework provided by Cunningham *et al*^15^ helps to structure the rather complex topic of evaluation of healthcare networks, but we found some limitations in its use. For instance, it does not provide an avenue to explore why the choice of a network design was considered appropriate; we would advocate examining the reasons for implementing networks in healthcare (as opposed to other potential organisational forms) alongside intended goals. Though staffing and budgetary control are factored into the framework, the long-term financing of networks is not. Resource mobilisation is crucial for LLMICs and an explicit assessment of how governments intend to finance primary care networks in the long run is important, alongside consideration of the role of external funding agencies. Additionally, the evaluation framework does not include an explicit ‘challenges’ section, which would support evaluators to explore difficulties surrounding implementation of incentive arrangements,^45^ long-term financial sustainability^46^ and capacity building for effective health workforce-community collaboration.^47^ Though these topics may arguably be explored under the effectiveness criteria, the importance of financial and workforce capacity issues in LLMICs requires them to be explored separately and in more depth.

### Study limitations

Our scoping review has a few limitations. First, we employ a structured methodology in screening for and evaluating the evidence of primary care networks in LLMICs, which provides strength to the review. However, there were limitations in retrieving relevant literature for PCNs in specific countries. An inclusion of an advisory committee from a range of countries to provide context-specific experiential knowledge and documentation may have strengthened the study further.

Second, the absence of a global definitive definition and characterisation of primary care networks has further limited the ability of our review to thoroughly identify and analyse relevant networks being implemented at the primary care level in LLMICs.

Third, our chosen focus of LLMICs may have limited the breadth of evidence available on PCNs. However, such a broad focus could likely have different implications to the study overall especially due to the vast heterogeneity across country income groups.

Finally, we would argue that the specific contextual conditions and challenges affecting LLMICs such as reliance on external donors, relative lack of resources in health systems and limited qualified healthcare staff per head of population mean that lessons cannot straightforwardly be transferred between the use of networks as an organisational form in LLMIC and their use in more resource-intensive settings.

## Conclusion

We identified several examples of PCNs in LLMICs and applied an overarching evaluation framework to the selected studies to map how primary care networks in LLMICs have been conceptualised in the literature. Through this process, we identified some significant evidence gaps. First, only a few studies addressed the motivation to choose a networked form of primary care provision over other available models. This is important, because the potential for service improvement will depend on the pre-existing system and level of service, and it is crucial to understand exactly what gaps or deficits the network approach is intended to address, and the mechanisms by which this is expected to occur. Second, the term ‘network’ has been used imprecisely and inconsistently, with many studies not clearly identifying the features of the system which make it a network rather than tilting more to hierarchy or another governance mode. Third, we found very limited evidence on the long-term effectiveness of networks, and on the extent to which the preconditions for effective networks such as trust, shared goals and legitimacy are met.

Future research work could shed light on some of these missing pieces of evidence on the effectiveness of PCNs in LLMIC, for example, considering differential consequences of network establishment and operation, including unintended consequences in the systems within which they reside, and evaluating long-term implications. From a policy perspective, these results call for a more thoughtful design and implementation of PCNs in LLMICs, building on a clear idea of what benefits are expected to accrue from a networked delivery of primary healthcare, by which mechanisms. Future PCNs in LLMICs should also outline clearer mechanisms to involve communities, and to resolve the potential conflicts between goals of non-governmental actors and government-mandated networks involving them. Finally, PCNs should be designed with clearly stated goals and measurable outcomes to facilitate evaluation.

## Data Availability

Data sharing not applicable as no data sets generated and/or analysed for this study. All data relevant to the study are included in the article or uploaded as supplementary information.
