# Supplementary material for: Evaluating primary care networks in low-income and lower middle-income countries: a scoping review
Source: BMJ Glob Health. 2023 Aug 14;8(8):e012505. doi: 10.1136/bmjgh-2023-012505 (PMC10432626; doi:10.1136/bmjgh-2023-012505)
Supplement: Supplementary data [file bmjgh-2023-012505supp001.pdf]

1.1. Supplementary File 1: Search Strategy

Database	Search details	Results (April 2022)	Results (November 2022)	Results (May 2023)	Total Results
Medline (PubMed)	((("afghan*"[All Fields] OR "angola*"[All Fields] OR "algeria*"[All Fields] OR "bangladesh*"[All Fields] OR "belize*"[All Fields] OR "benin*"[All Fields] OR "bhutan*"[All Fields] OR "boliv*"[All Fields] OR ("Burkina"[All Fields] AND "faso*"[All Fields]) OR "burundi*"[All Fields] OR (("cabo"[Journal] OR "cabo"[All Fields]) AND "verde*"[All Fields]) OR ("Cape"[All Fields] AND "verde*"[All Fields]) OR "cambod*"[All Fields] OR "cameroon*"[All Fields] OR "cameroun*"[All Fields] OR ("central african republic"[MeSH Terms] OR ("central"[All Fields] AND "african"[All Fields] AND "republic"[All Fields]) OR "central african republic"[All Fields]) OR "chad*"[All Fields] OR "comoro*"[All Fields] OR "congo*"[All Fields] OR ("cote d ivoire"[MeSH Terms] OR ("cote"[All Fields] AND "d ivoire"[All Fields]) OR "cote d ivoire"[All Fields]) OR "djibouti*"[All Fields] OR "egypt*"[All Fields] OR ("El"[All Fields] AND "salvador*"[All Fields]) OR "eritre*"[All Fields] OR "eswatini*"[All Fields] OR "ethiopia*"[All Fields] OR "gambia*"[All Fields] OR "gaza*"[All Fields] OR "ghan*"[All Fields] OR "guinea*"[All Fields] OR "haiti*"[All Fields] OR "hondur*"[All Fields] OR "india*"[All Fields] OR "indones*"[All Fields] OR "iran*"[All Fields] OR (("ivories"[All Fields] OR "ivory"[All Fields]) AND "coast*"[All Fields]) OR "kenya*"[All Fields] OR "kiribati*"[All Fields] OR "kyrgyz*"[All Fields] OR "lao"[All Fields] OR "lesotho*"[All Fields] OR "liberia*"[All Fields] OR "madagascar*"[All Fields] OR "malaw*"[All Fields] OR "mali*"[All Fields] OR "mauritan*"[All Fields] OR "micronesi*"[All Fields] OR "mocambiqu*"[All Fields] OR "mongolia*"[All Fields] OR "morocc*"[All Fields] OR "mozambiqu*"[All Fields] OR "myanmar*"[All Fields] OR "nepal*"[All Fields] OR "nicaragua*"[All Fields] OR "niger*"[All Fields] OR (("northern"[All Fields] OR "northerners"[All Fields] OR "northerns"[All Fields]) AND "korea*"[All Fields]) OR (("north"[All Fields] OR "norths"[All Fields]) AND "korea*"[All Fields]) OR "pakistan*"[All Fields] OR ("Papua"[All Fields] AND "New"[All Fields] AND "guinea*"[All Fields]) OR "philippine*"[All Fields] OR "principe*"[All Fields] OR "korea*"[All Fields] OR "rwanda*"[All Fields] OR "samoa*"[All Fields] OR ("Sao"[All Fields] AND "tome*"[All Fields]) OR "senegal*"[All Fields] OR (("sierra"[All Fields] OR "sierras"[All Fields]) AND "leone*"[All Fields]) OR (("solomon"[All Fields] OR "solomons"[All Fields]) AND "islands*"[All Fields]) OR "somalia*"[All Fields] OR ("South"[All Fields] AND "africa*"[All Fields]) OR "sudan*"[All Fields] OR ("Sri"[All Fields] AND "lanka*"[All Fields]) OR "sudan*"[All Fields] OR "syria*"[All Fields] OR "tajikist*"[All Fields] OR "tanzan*"[All Fields] OR "timor*"[All Fields] OR "togo*"[All Fields] OR "tunis*"[All Fields] OR "ugand*"[All Fields] OR "ukrain*"[All Fields] OR ("uzbekistan"[MeSH Terms] OR "uzbekistan"[All Fields])) AND "vanuatu*"[All Fields]) OR "vietnam*"[All Fields] OR ("West"[All Fields] AND "bank*"[All Fields]) OR "yemen*"[All Fields] OR "zaire*"[All Fields] OR "zambia*"[All Fields] OR "zimbabw*"[All Fields])<br><br>AND<br><br>(("primary health care"[MeSH Terms] OR ("primary"[All Fields] AND "health"[All Fields] AND "care"[All Fields]) OR "primary	4042	64	-	4106

	health care"[All Fields]) OR ("community"[All Fields] AND "health"[All Fields] AND "care"[All Fields]) OR "community health care"[All Fields]) AND ("network"[All Fields] OR "network s"[All Fields] OR "networked"[All Fields] OR "networker"[All Fields] OR "networkers"[All Fields] OR "networking"[All Fields] OR "networks"[All Fields])				
Embase (Ovid)	((Afghan$ or Angola$ or Algeria$ or Bangladesh$ or Belize$ or Benin$ or Bhutan$ or Boliv$ or Burkina Faso$ or Burundi$ or Cabo Verde$ or Cape Verde$ or Cambod$ or Cameroon$ or Cameroun$ or Central African Republic or Chad$ or Comoro$ or Congo$ or Cote d Ivoire or Djibouti$ or Egypt$ or El Salvador$ or Eritre$ or Eswatini$ or Ethiopia$ or Gambia$ or Gaza$ or Ghan$ or Guinea$ or Haiti$ or Hondur$ or India$ or Indones$ or Iran$ or Ivory Coast$ or Kenya$ or Kiribati$ or Kyrgyz$ or Lao$ or Lesotho$ or Liberia$ or Madagascar$ or Malaw$ or Mali$ or Mauritan$ or Micronesi$ or Mocambiqu$ or Mongolia$ or Morocc$ or Mozambiqu$ or Myanmar$ or Nepal$ or Nicaragua$ or Niger$ or Northern Korea$ or North Korea$ or Pakistan$ or Papua New Guinea$ or Philippine$ or Principe$ or Korea$ or Rwanda$ or Samoa$ or Sao Tome$ or Senegal$ or Sierra Leone$ or Solomon Islands$ or Somalia$ or South Africa$ or Sudan$ or Sri Lanka$ or Sudan$ or Syria$ or Tajikist$ or Tanzan$ or Timor$ or Togo$ or Tunis$ or Ugand$ or Ukrain$ or Uzbekistan& or Vanuatu$ or Vietnam$ or West Bank$ or Yemen$ or Zaire$ or Zambia$ or Zimbabw$)<br>AND<br>(primary healthcare or primary care or primary health care) and network).mp. [mp=title, abstract, heading word, drug trade name, original title, device manufacturer, drug manufacturer, device trade name, keyword heading word, floating subheading word, candidate term word]	595	62	-	657
Health Management Information Consortium (HMIC) (Ovid)	((Afghan$ or Angola$ or Algeria$ or Bangladesh$ or Belize$ or Benin$ or Bhutan$ or Boliv$ or Burkina Faso$ or Burundi$ or Cabo Verde$ or Cape Verde$ or Cambod$ or Cameroon$ or Cameroun$ or Central African Republic or Chad$ or Comoro$ or Congo$ or Cote d Ivoire or Djibouti$ or Egypt$ or El Salvador$ or Eritre$ or Eswatini$ or Ethiopia$ or Gambia$ or Gaza$ or Ghan$ or Guinea$ or Haiti$ or Hondur$ or India$ or Indones$ or Iran$ or Ivory Coast$ or Kenya$ or Kiribati$ or Kyrgyz$ or Lao$ or Lesotho$ or Liberia$ or Madagascar$ or Malaw$ or Mali$ or Mauritan$ or Micronesi$ or Mocambiqu$ or Mongolia$ or Morocc$ or Mozambiqu$ or Myanmar$ or Nepal$ or Nicaragua$ or Niger$ or Northern Korea$ or North Korea$ or Pakistan$ or Papua New Guinea$ or Philippine$ or Principe$ or Korea$ or Rwanda$ or Samoa$ or Sao Tome$ or Senegal$ or Sierra Leone$ or Solomon Islands$ or Somalia$ or South Africa$ or Sudan$ or Sri Lanka$ or Sudan$ or Syria$ or Tajikist$ or Tanzan$ or Timor$ or Togo$ or Tunis$ or Ugand$ or Ukrain$ or Uzbekistan& or Vanuatu$ or Vietnam$ or West Bank$ or Yemen$ or Zaire$ or Zambia$ or Zimbabw$)<br>AND<br>(primary healthcare or primary care or primary health care) and network).mp. [mp=title, abstract, heading word, drug trade	-	-	6	6

	name, original title, device manufacturer, drug manufacturer, device trade name, keyword heading word, floating subheading word, candidate term word]				
Global Health Archive (Ovid)	((Afghan$ or Angola$ or Algeria$ or Bangladesh$ or Belize$ or Benin$ or Bhutan$ or Boliv$ or Burkina Faso$ or Burundi$ or Cabo Verde$ or Cape Verde$ or Cambod$ or Cameroon$ or Cameroun$ or Central African Republic or Chad$ or Comoro$ or Congo$ or Cote d Ivoire or Djibouti$ or Egypt$ or El Salvador$ or Eritre$ or Eswatini$ or Ethiopia$ or Gambia$ or Gaza$ or Ghan$ or Guinea$ or Haiti$ or Hondur$ or India$ or Indones$ or Iran$ or Ivory Coast$ or Kenya$ or Kiribati$ or Kyrgyz$ or Lao$ or Lesotho$ or Liberia$ or Madagascar$ or Malaw$ or Mali$ or Mauritan$ or Micronesi$ or Mocambiqu$ or Mongolia$ or Morocc$ or Mozambiqu$ or Myanmar$ or Nepal$ or Nicaragua$ or Niger$ or Northern Korea$ or North Korea$ or Pakistan$ or Papua New Guinea$ or Philippine$ or Principe$ or Korea$ or Rwanda$ or Samoa$ or Sao Tome$ or Senegal$ or Sierra Leone$ or Solomon Islands$ or Somalia$ or South Africa$ or Sudan$ or Sri Lanka$ or Sudan$ or Syria$ or Tajikist$ or Tanzan$ or Timor$ or Togo$ or Tunis$ or Ugand$ or Ukrain$ or Uzbekistan& or Vanuatu$ or Vietnam$ or West Bank$ or Yemen$ or Zaire$ or Zambia$ or Zimbabw$)<br><br>AND<br><br>(primary healthcare or primary care or primary health care) and network).mp. [mp=title, abstract, heading word, drug trade name, original title, device manufacturer, drug manufacturer, device trade name, keyword heading word, floating subheading word, candidate term word]	-	-	3	3
