# Supplementary material for: Evaluating primary care networks in low-income and lower middle-income countries: a scoping review
Source: BMJ Glob Health. 2023 Aug 14;8(8):e012505. doi: 10.1136/bmjgh-2023-012505 (PMC10432626; doi:10.1136/bmjgh-2023-012505)
Supplement: Supplementary data [file bmjgh-2023-012505supp002.pdf]

Supplementary File 2

Websites searched for grey literature.

https://www.salud.gob.hn/sshome/

https://www.minisanterdc.cd/

https://irangov.ir/ministry-of-health-and-medical-education

http://health.gov.ls/

https://www.minsalud.gob.bo/

https://scholar.google.com/

https://www.worldbank.org/en/research?cid=ECR_GA_worldbank_EN_EXTP_search&s_kwcid=AL!18468!3!610032451099!b!!g!!what%20does%20the%20world%20bank%20do&gclid=CjwKCAjwp6CkBhB_EiwAlQVyxYtuWhMtNwc0ZREoHGMSUFVI8ArQOAqz7Yez2z9bE_0yCW_M-porpRoCaaMQAvD_BwE

https://www.who.int/health-topics/research#tab=tab_1

www.improvingphc.org
