# Supplementary material for: Evaluating primary care networks in low-income and lower middle-income countries: a scoping review
Source: BMJ Glob Health. 2023 Aug 14;8(8):e012505. doi: 10.1136/bmjgh-2023-012505 (PMC10432626; doi:10.1136/bmjgh-2023-012505)
Supplement: Supplementary data [file bmjgh-2023-012505supp003.pdf]

1.1. Supplementary File 3: Description of Documents

No	Country	Reference	Year	Document type	Sample size	Aim of study/document	Methods
1	Iran	Bagheri, Z., Dehdari, T., & Lotfizadeh, M. (2021). The Preparedness of Primary Health Care Network in terms of Emergency Risk Communication: A Study in Iran. Disaster Medicine and Public Health Preparedness, 1–10. https://doi.org/10.1017/dmp.2021.70	2021	Journal Article	136 Primary Health Care Facilities	To investigate preparedness of the Primary Health Care Network (PHCN) of Iran in terms of Emergency Risk Communication (ERC)	Cross-sectional study: Emergency risk communication data collected with Centre of Disease Control and Prevention (CDC) checklist and analysed
2		Takian, A., Doshmangir, L., & Rashidian, A. (2013). Implementing family physician programme in rural Iran: Exploring the role of an existing primary health care network. Family Practice, 30(5), 551–559. https://doi.org/10.1093/fampra/cmt025	2013	Journal Article	71 semi-structured interviews, three focus group discussions	To explore the role of pre-existing PHC network on the implementation of a family planning programme in rural Iran	Interviews at national, provincial and local levels, and focus group discussions at local level, content analysis of documents
3		Verulava, T. (2006). Health care system in the Islamic Republic of Iran. Insurance. Health Policy & Management.	2006	Journal Article:	N/A	N/A	N/A
4		Tabrizi, J.S., Pourasghar, F, & Gholamzadeh Nikjoo, R. (2017). Status of Iran's Primary Health Care System in Terms of Health	2017	Journal Article: Review of	N/A	To assess the status of Iran's PHC system (strengths, weaknesses, opportunities and threats)	Database searches in Persian and English with no time limits.

		Systems Control Knobs: A Review Article. Iranian Journal of Public Health, 46(9), 1156–1166.		literature		in terms of health system's control knobs	
5		Mehrdad, R. (2009). Health system in Iran. JMAJ, 52(1), 69–73.	2009	Journal Article: Commentary	N/A	N/A	N/A
6		Sajadi, H. S., & Majdzadeh, R. (2019). From Primary Health Care to Universal Health Coverage in the Islamic Republic of Iran: A Journey of Four Decades. Archives of Iranian Medicine, 22(5), 262–268.	2019	Journal Article	N/A	N/A	N/A
7		Yazdi-Feyzabadi, V., Bazyar, M., & Ghasemi, S. (2021). District health network policy in Iran: The role of ideas, interests, and institutions (3i framework) in a nutshell. Archives of Public Health, 79(1), 212. https://doi.org/10.1186/s13690-021-00737-7	2021	Journal Article: Review of literature	N/A	To use interrelated elements of the 3i framework to explain retrospectively how the district health network of Iran was formed	Database search and extraction of secondary source data, supplemented with primary interviews
8		Yazdi-Feyzabadi, V., Delavari, S., & Ghasemi, S. (2018). Viewpoint: Primary care in Iran needs a paradigm shift. British Journal of General Practice, 68(670), 235–235. https://doi.org/10.3399/bjgp18X696005	2018	Journal Article: Opinion Piece	N/A	N/A	N/A

9		Yazdi-Feyzabadi, V., Emami, M., & Mehrolhassani, M. (2015). Health information system in primary health care: The challenges and barriers from local providers' perspective of an area in Iran. International Journal of Preventive Medicine, 6(1), 57–57.	2015	Journal Article: Primary Research	2 focus group discussions	To investigate PHC providers' perspectives on health information system	Qualitative approach of semi-structured audiotaped focus group discussions. Framework analysis method used.
10		Ghorrabi AT, Kakemam E, Moradi-Joo E, Dehcheshmeh NF. Challenges of the organizational structure of county health network in Iran: findings from a qualitative study. BMC Health Serv Res. 2022 May 28;22(1):712. doi: 10.1186/s12913-022-08104-0. PMID: 35643548; PMCID: PMC9145099.	2022	Journal Article: Primary Research	21 semi-structured interviews in Ahvaz	To investigate and identify challenges of the organisational structure of a health network and provide solutions to eliminate and/or correct these	Qualitative study with thematic analysis. Interveiwees were key informants, managers and experts from Ahvaz Jundishapur University of Medical Sciences
11		Bazyar M, Yazdi-Feyzabadi V, Bahmani M, Sadeghifar J, Momeni K, Shaabani Z. (2022) Preferences of people in choosing a family physician in rural areas: a qualitative inquiry from Iran. Primary Health Care Research & Development 23(e57): 1–10. doi: 10.1017/S1463423622000317	2022	Journal Article: Primary Research	34 interviews (unstructured and semi-structured) in one province	To identify influencing factors for choosing a new family physician or changing current one	Qualitative study with thematic analysis. Interviews conducted with officials of district health network, patients, family physicians and insurance staff at Ilam province
12	Lesotho	McIntosh, N., Grabowski, A., Jack, B., Nkabane-Nkholongo, E. L., & Vian, T. (2015). A Public-Private Partnership Improves Clinical	2015	Journal Article: Primary	8 quantitative measures collected at baseline and	To compare government-managed healthcare network with PPP-managed network that	Mixed methods stud, quantitative approach to compare baseline and end-line measures,

		Performance In A Hospital Network In Lesotho. Health Affairs, 34(6), 954-962,1-6. http://dx.doi.org/10.1377/hlthaff.2014.0945		Research	end-line. 36 semi-structured interviews	replaced it.	qualitative approach of semi-structured interviews of key informants of PPP
13		World Bank. (2016, February 19). Lesotho Health Network Public-Private Partnership (PPP) [Text/HTML]. World Bank. https://www.worldbank.org/en/country/lesotho/brief/lesotho-health-network-ppp	2016	Webpage: Brief	N/A	N/A	N/A
14		Webster, P. C. (2015). Lesotho's controversial public–private partnership project. The Lancet, 386(10007), 1929–1931. https://doi.org/10.1016/S0140-6736(15)00959-9	2015	Journal Article: Report	N/A	N/A	N/A
15		Vian, T., McIntosh, N., Grabowski, A., Brooks, B., Jack, B., & Nkabane-Nkholongo, E. (2013). Endline study for Queen 'Mamohato Hospital public private partnership (PPP). Washington, DC: World Bank.	2013	Project Report	N/A	N/A	N/A
16	DRC	Bwimana, A. (2017). Heath Sector Network Governance and State-building in South Kivu, Democratic Republic of Congo. Health Policy and Planning, 32(10), 1476–1483.	2017	Journal Article: Primary Research	415 open and semi-structured interviews, 34 focus group	To explore governance networks in DRC's health sector	Qualitative study with interviews, focus groups, direct and indirect observations

		https://doi.org/10.1093/heapol/czx095			discussions		
17		World Bank. (2005). Democratic Republic of Congo: Health, Nutrition and Population, Country Status Report. World Bank. https://openknowledge.worldbank.org/handle/10986/8771	2005	Report	N/A	N/A	N/A
18	Bolivia	Lavadenz, F., Schwab, N., & Straatman, H. (2001). [Public, decentralized and community health networks in Bolivia]. Revista Panamericana De Salud Publica = Pan American Journal of Public Health, 9(3), 182–189. https://doi.org/10.1590/s1020-49892001000300008	2001	Journal Article	N/A	N/A	N/A
19	Honduras	Puertas, E. B., Martínez, R. A., Figueroa, G. S., & Hidalgo, F. E. (2018). Integración de redes de servicios de salud en Honduras: Valoración comparativa del planteamiento teórico y de la aplicación práctica en cinco redes del país. Revista Panamericana de Salud Pública, 42, e135. https://doi.org/10.26633/RPSP.2018.135	2018	Journal Article	6 official documents, 5 healthcare networks	To assess and compare the development of integrated health service delivery networks (IHSDNs) in 5 networks	Theoretical assessment of documents and practical assessments of networks, both with the IHSDB Assessment tool of the Pan American Health Organization (PAHO)

20		Carmenate-Milián, L., Alej, Herrera-Ramos, R., Ramos-Cáceres, D., Ordoñez, K. L.-, Ordoñez, T. L.-, & Somoza-Valladares, C. (2017). Situation of the Health System in Honduras and the New Proposed Health Model. *Archives of Medicine*, *9*(4), 0–0. https://doi.org/10.21767/1989-5216.1000222	2017	Journal Article	N/A	To describe healthcare system in Honduras and propose New Model of Care	Systematic Review
